# Tularemia Outbreak, Bulgaria, 1997–2005

**DOI:** 10.3201/eid1204.050709

**Published:** 2006-04

**Authors:** Todor Kantardjiev, Ivan Ivanov, Tzvetan Velinov, Plamen Padeshki, Boris Popov, Roumiana Nenova, Milcho Mincheff

**Affiliations:** * National Center for Infectious and Parasitic Diseases, Sofia, Bulgaria;; † The George Washington University Medical Center, Washington, DC, USA

**Keywords:** *Francisella tularensis*, tularemia outbreak, AFLP typing, dispatch

## Abstract

The 1997–2005 tularemia outbreak in Bulgaria affected 285 people. Ten strains were isolated from humans, a tick, a hare, and water. Amplified fragment length polymorphism typing of the present isolates and of the strain isolated in 1962 suggests that a new genetic variant caused the outbreak.

Tularemia is a zoonotic disease caused by the gram-negative bacterium *Francisella tularensis* (*1*). During the last 10 years, several outbreaks occurred in different countries, causing tularemia to become a major problem on the Balkan Peninsula (*2**–**5*).

The first Bulgarian *F. tularensis* strain, isolated in 1962 from a muskrat (*Ondatra zibethica*) found in the lake of Srebarna reserve near the Danube River, was designated Srebarna19 (*6*). The first 4 tularemia cases in Bulgaria were reported in 1963 (*6**,**7*) after a small epidemic involving mostly employees in the Srebarna reserve. After 35 years of tularemia surveillance with no cases reported, a focal epidemic was detected near the end of 1997 (*2**,**8*). New cases appeared, and strains were isolated and characterized. A total of 285 cases of tularemia were reported and registered at the Bulgarian Ministry of Health in the period 1997–2004 and the first quarter of 2005. The outbreak areas in 1962 and 1997–2005 in Bulgaria are shown on Figure 1. The first case of tularemia was reported in November 1997 in a patient from a small town in the Slivnitsa region. From 1998 to 2000, 171 cases were reported (*8*). The outbreak seemed to abate during 2001 and 2002, when only 16 cases were documented. The incidence increased again in 2003 when 76 new cases were reported. An area ≈4,000 km^2^ near the western border with Serbia and Montenegro was the epidemic focus of the outbreak.

**Figure 1:**
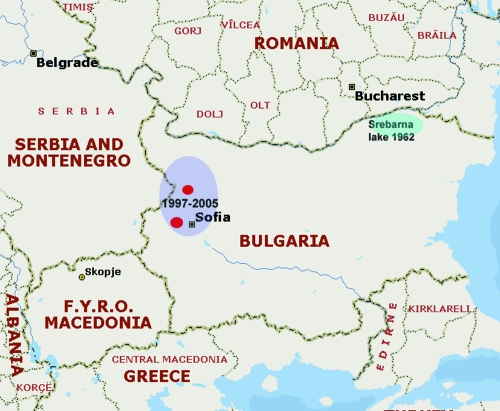
The outbreak areas, Bulgaria, 1962 and 1997–2005.

All the patients exhibited the typical clinical picture of oropharyngeal, oculoglandular, or ulceroglandular tularemia. Four (1.4%) of the 285 patients had the oculoglandular form, 6 (2.1%) had the ulceroglandular form, and 275 (96.5%) had the oropharyngeal form. No deaths, complications, or relapses were observed.

Except for 1 seronegative patient, tularemia cases were diagnosed according to the confirmed case definition of the Centers for Disease Control and Prevention (*9*). Clinically relevant information was gathered by interviews, referral to hospitals, and questionnaires sent to general practitioners in the region and submitted to the reference centers for epidemiologic analysis. Three serum samples (acute phase, convalescent phrase, and l collected 3 months ± 15 days after the end of therapy) were collected from all patients (Table A1). All samples were tested with hemagglutination and tube-agglutination assays (BulBio-NCIPD, Sofia, Bulgaria) for anti-*Francisella* antibodies.

Fine needle biopsy specimens from enlarged lymph nodes were processed from 20 patients. Half of the volume from each specimen was cultured on modified Thayer-Martin agar (*10*), and the other half was processed for polymerase chain reaction (PCR). Water samples, collected from 41 wells, were also cultured through passage in guinea pigs (Appendix). Ten strains were isolated, 4 from patients, 4 from water, 1 from a hare, and 1 from a tick (Appendix). One of the human isolates (isolate Las) was from a seronegative patient. Identification of the strains was performed according to their microbiologic and antigenic properties by using standard methods (*10*). Direct immunofluorescence assay (IFA) with fluorescein isothiocyanate–conjugated anti-*Francisella* serum (BulBio-NCIPD) was used to detect *F. tularensis* antigens. DNA from biopsy specimens and strains was subjected to PCR with tul4 and RD1 primers (*11*). All investigated biopsy specimens and strains were PCR and IFA positive.

16S-PCR restriction fragment length polymorphisms (RFLP) and amplified fragment length polymorphism (AFLP) methods were used for molecular typing. For 16S-PCR RFLP, the genomic DNA was amplified by 16S rRNA universal primers (*12*). The 948-bp PCR product was digested with *Mbo*I, *Rsa*I, and *Hae*III enzymes. All strains exhibited a characteristic *F. tularensis* fingerprinting pattern, and no variations were found. For AFLP, DNA was digested with *Hind*III and *Mbo*I enzymes, adaptors were ligated, and selective PCR was carried out with Hind+0 and Mbo+C primers. Pearson correlation and unweighted pair group method with arithmetic averages (UPGMA) algorithms were applied for generating dendrogram (Figure 2). Three of the water isolates showed 100% similarity, and only 1 (Aqua D) was included in the dendrogram (Figure 2). A set of DNA samples from 27 *F. tularensis* strains originating from Asia, Europe, America, and Bulgaria were also typed. The AFLP method clearly discriminated the representatives of different phylogenetic *F. tularensis* groups. Although the 27 AFLP patterns show little variability (<25%, Figure 2), distinctive clusters are seen. All of the subspecies *holarctica* cluster away from the subspecies *tularensis*. The fingerprinting pattern of a strain Srebarna19, isolated in 1962 during an outbreak near the Lake of Srebarna, shows high similarity with fingerprints of strains isolated in Europe (e.g., the 335–64, Italy 1964, Figure 2). The dendrogram clearly shows divergence between the 1962 Srebarna19 strain and the organisms associated with the current outbreak. The human, water, and animal isolates from the current outbreak have ≈95% similarity. The human isolates are closely related to isolates recovered from well water but are more distantly related to isolates from the hare and tick. The AFLP data of isolates from the current outbreak support the hypothesis of a new genotype emerging in Bulgaria. AFLP also shows the emerging isolates to be genetically distinct from the European, Asian, and American isolates evaluated in this study.

**Figure 2:**
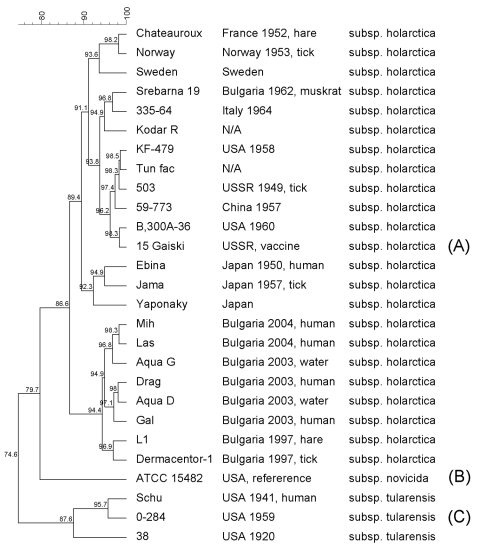
Amplified fragment length polymorphism dendrogram.

Several publications describe outbreaks in the Balkan Peninsula with healthcare importance (*3**–**5*). Ten *F. tularensis* strains were isolated in Turkey (*3*). However, they were not genetically characterized. No isolation was attempted during the Kosovo outbreak in 1999 (*5*).

The origin of the 10 strains from the current outbreak is controversial, but they are clearly distinct from the other worldwide isolates included in our study. The new outbreak may be a result of the agricultural reorganizations in Bulgaria in 1990s. These changes affected the way in which the arable soil was ploughed, leaving rodent holes intact. As a result, the populations of rodents, considered the main reservoir of the infection, increased substantially (*13*).

*Francisella* organisms can survive in water for prolonged periods, probably by interaction with protozoa (*14*). The isolation of bacteria from 4 private wells in the affected area points to ingestion of contaminated food or drinking water as the probable route of infection. This finding is further supported by the observation that most of the cases represent the oropharyngeal form. Rodents (or their excrement) could be the source for water contamination, but this hypothesis is not confirmable because of the lack of later rodent isolates for comparison.

The organism might have been introduced by means of rodents and hares through the border with Serbia and Montenegro. Agricultural practices are alike in the neighboring countries, and a similar boom in the rodent population might also have occurred there. Such a migration is bidirectional, but a future collaborative study with colleagues from Serbia and Macedonia, where tularemia is also problematic, is necessary to answer this question. Typing isolates originating from different Balkan countries will show the genetic relatedness and biodiversity among resident *F. tularensis* populations.

The cases reported in 2004 and 2005 suggest that the outbreak is still in progress. These are the first data for genetic identification and typing of isolates from the Balkan region, and they show a new genotype of *F. tularensis* emerging as a cause of human disease in Bulgaria.

## Appendix

### Microbiologic and Epidemiologic Approaches for Investigation of Outbreak

#### Epidemiology

After the 1962 outbreak, annual active epidemiologic surveillance was carried out through a plan worked out by the public health authorities and included the following: 1) bacteriologic examination of trapped and dead rodents, 2) bacteriologic examination of water samples, and 3) serologic examination of patients with unexplainable enlarged lymph nodes.

A total of 285 patients had tularemia from 1997 to 2005. All patients were initially interviewed by a primary care physician, who sent the patients to the Hospital for Infectious Diseases (upon tularemia indication) and the results to the Regional Epidemiology Center. The S1 serum samples were collected immediately after clinical indication (1–4 weeks after the onset of symptoms) and the S2 (convalescent-phase) serum samples were collected 15 ±2 days after the S1. S3 samples were collected 3 months ±15 days after the end of therapy.

The epidemiologic survey included the following information: address, onset of disease, place of work and possible contact with animals, presence of dust at the work place, water and food sources, hunting or consumption of game, and history of tick bite. Questionnaires filled out by the patients show that they did not drink water from the contaminated wells. The water, however, was used for irrigating backyard vegetable gardens, and those vegetables were eaten. Since the human isolates are closely related to isolates recovered from well water and less to isolates from hares and ticks, we consider the alimentary route from food by contaminated rodents or water as the principal one.

#### Culture Methods

Five hundred milliliters of water were collected in a sterile container. All subsequent steps were carried out in a class III biosafety cabinet. The water was centrifuged at 5,000 × *g* for 10 min. The pellet was dissolved in 1.5 mL sterile phosphate-buffered saline (PBS) and centrifuged again as above. The pellet was again dissolved in 1.5 mL PBS. One third (0.5 mL) was cultured on modified Thayer-Martin agar. Guinea pigs were passed intraperitoneally with another 0.5 mL, and the remaining 0.5 mL was kept at –70°C. Guinea pigs were operated on at day 7 or at the day of exitus. Cultures were made from a homogenized spleen and liver suspension. Four strains were isolated by the passage method (Table A1).

## Appendix

**Table A1:** Time, source, and origin of isolated tularemia strains

Isolate, source	Year	Village	Region
Dermacentor-1, tick	1997	Aldomirovci	Slivnitsa
L1, hare	1997	Aldomirovci	Slivnitsa
Gal, human	2003	Meshtica	Pernik
Drag, human	2003	Meshtica	Pernik
Aqua D, well water	2003	Meshtica	Pernik
Aqua G, well water	2003	Meshtica	Pernik
Aqua A, well water	2003	Meshtica	Pernik
Aqua A, well water	2003	Meshtica	Pernik
Las, human	2004	Aldomirovci	Slivnitsa
Mih, human	2004	Aldomirovci	Slivnitsa
